# Regulation of Gene Expression in Hepatic Cells by the Mammalian Target of Rapamycin (mTOR)

**DOI:** 10.1371/journal.pone.0009084

**Published:** 2010-02-05

**Authors:** Rosa H. Jimenez, Ju-Seog Lee, Mirko Francesconi, Gastone Castellani, Nicola Neretti, Jennifer A. Sanders, John Sedivy, Philip A. Gruppuso

**Affiliations:** 1 Department of Pediatrics, Rhode Island Hospital and Brown University, Providence, Rhode Island, United States of America; 2 Molecular Therapeutics, University of Texas MD Anderson Cancer Center, Houston, Texas, United States of America; 3 Interdepartmental Center “L. Galvani”, Bologna University, Bologna, Italy; 4 Institute for Brain and Neural Systems, Brown University, Providence, Rhode Island, United States of America; 5 Department of Molecular Biology, Cell Biology and Biochemistry and Center for Genomics and Proteomics, Brown University, Providence, Rhode Island, United States of America; University of Washington, United States of America

## Abstract

**Background:**

We investigated mTOR regulation of gene expression by studying rapamycin effect in two hepatic cell lines, the non-tumorigenic WB-F344 cells and the tumorigenic WB311 cells. The latter are resistant to the growth inhibitory effects of rapamycin, thus providing us with an opportunity to study the gene expression effects of rapamycin without confounding effects on cell proliferation.

**Methodology/Principal Findings:**

The hepatic cells were exposed to rapamycin for 24 hr. Microarray analysis on total RNA preparations identified genes that were affected by rapamycin in both cell lines and, therefore, modulated independent of growth arrest. Further studies showed that the promoter regions of these genes included E-box-containing transcription factor binding sites at higher than expected rates. Based on this, we tested the hypothesis that c-Myc is involved in regulation of gene expression by mTOR by comparing genes altered by rapamycin in the hepatic cells and by c-Myc induction in fibroblasts engineered to express c*-myc* in an inducible manner. Results showed enrichment for c-Myc targets among rapamycin sensitive genes in both hepatic cell lines. However, microarray analyses on wild type and c-*myc* null fibroblasts showed similar rapamycin effect, with the set of rapamycin-sensitive genes being enriched for c-Myc targets in both cases.

**Conclusions/Significance:**

There is considerable overlap in the regulation of gene expression by mTOR and c-Myc. However, regulation of gene expression through mTOR is c-Myc-independent and cannot be attributed to the involvement of specific transcription factors regulated by the rapamycin-sensitive mTOR Complex 1.

## Introduction

The mammalian Target of Rapamycin (mTOR) is a central regulator of many biological processes that are essential for cell growth, including cell cycle progression, protein translation, ribosomal biogenesis, autophagy and metabolism [1], [2]. In addition, alterations in mTOR signaling have been associated with the pathophysiology of cancer [3], [4] and aging [5]. Among the effects of mTOR signaling, regulation of gene expression and transcription is well characterized for several specific genes, including DNA polymerase I (Pol I), Insulin Growth Factor II (IGF II), ribosomal DNA [6]–[8] and regulators of mitochondrial oxidative function [9]. However, general effects on gene expression have not been well characterized, and the mechanisms by which mTOR signaling affects the expression of a broad array of genes are less well defined than is the case for its other biological effects.

mTOR interacts with a number of protein partners, including raptor (regulatory associated protein of TOR) and rictor (rapamycin-insensitive companion of TOR), which form complexes with mTOR termed mTORC1 and mTORC2, respectively [10]. mTORC1-mediated signaling events are sensitive to the macrolytic lactone, rapamycin, while events downstream from mTORC2 are, in general, rapamycin insensitive [11].

The role of TOR in the regulation of transcription has been best characterized in yeast [12] where the nuclear localization and the activity of several nutrient and stress-responsive transcription factors are regulated by TORC1-dependent phosphorylation [13]. Yeast TOR signals to a number of specific effectors including Tap42, Mks1p, Ure2p, Gln3p, Npr1, Tip41, and Gat1p, all of which can elicit changes in the expression levels of enzymes involved in metabolic pathways [14], [15]. As a nutrient-sensing kinase, TOR signaling regulates the subcellular localization of the so-called unconventional prefoldin RPB5 interactor (URI), which is involved in the regulation of nutrient-sensitive, TORC1-controlled transcription pathways in yeast and mammals [16].

Examples of rapamycin-sensitive mTORC1 signaling to transcriptional regulators have been identified in eukaryotes. mTOR phosphorylates the signal transducers and activators of transcription 1 and 3 (STAT1 and STAT3) and, as a result, induces the transcription of the nuclear receptor peroxisome proliferator-activated receptors-gamma (PPARγ) [13]. In addition, mTOR signaling occupies a central role in the global regulation of ribosomal RNA synthesis through the TIF-IA transcription factor [17]. Finally, mTOR-mediated regulation of mitochondrial oxidative function occurs through regulation of a transcription factor termed YY1 [9].

We have characterized the effects of rapamycin on gene expression in two related rat liver epithelial cell lines [18]. The slow growing, non-tumorigenic WB-F344 [19] cell line is sensitive to the anti-proliferative effects of rapamycin while the faster growing, tumorigenic WB311 cell line, spontaneously derived from the WB-F344 cell line [20], is not. We used microarray analysis to characterize the effects of rapamycin on gene expression in these two cell lines. The results identified 106 genes whose expression was altered in response to rapamycin in both cell lines and, therefore, affected independent of rapamycin-induced growth arrest. We hypothesized that this set of genes would be regulated through effects on specific transcription factors and, therefore, be suitable for further study aimed at identifying transcriptional control mechanisms downstream from mTORC1.

## Methods

### Cell Culture

Culture conditions for the WB-F344 [19] and WB311 [20] cell lines have been previously described. Conditions for maintaining the TGR-1 (*c-myc* ^+/+^) and HO15.19 (*c-myc* ^−/−^) cells have been published as well [21]. All cell lines were grown at 37°C with 5.0% CO_2_ and plated at densities providing for approximately 60 to 80 percent confluence at the time of each experiment. After plating, cells were allowed to recover for at least 18 hr before exposure to rapamycin.

### Identification of Predicted Transcription Factor Binding Sites

The TOUCAN 2 web based program [22], [23], which integrates several data and algorithmic resources including TRANSFAC, JASPAR, Ensembl, MotifScanner and MotifSearcher, was used to identify overrepresented transcription factor binding sites in the proximal promoters or the distal non-coding sequence in defined sets of co-regulated or co-expressed genes. For the retrieval of promoter sequences from the Ensemble database, the following settings were used: Get_Seq from Ensembl/Biomart, comma separated, rat species, AFFY_Rat230_2 chip, 5′Upstreatm Exon 1, 1,000 base pairs (bp) upstream, 200 bp downstream, add to current list, Rev Compl automatically. To search for transcription factor binding sites, MotifScanner was used with the following settings: motif Position Weight Matrix (PWM) scoring, Motif Scanner, P2: TRANSFAC 7.0, P3: Prior 0.1, P4: background model (mouse DBTSS promoters [3]), stand integer 1. Note that the background model for the rat was not available in the TOUCAN2 program. Therefore, we used mouse as the background model.

The TOUCAN2 analysis was followed by statistical analysis to identify overrepresented transcription factor binding sites. The following parameters were used: Expected Frequencies file: epd_mus_musculus_prior 0.1.freq. Note that the expected frequencies file was not available for the rat, so the file for mouse was used. The prior that was used was the same as for the MotifScanner run. The expected frequencies file, located on the TOUCAN FTP-site, is based on the Eukaryotic Promoter Database (http://www.epd.isb.ch/seq_download.html).

### Analysis of Myc Family Gene Expression

WB-F344 cells were plated in 100 mm plates and allowed to attach overnight. Vehicle (DMSO) or 50 nM rapamycin was added for 6 or 24 hr. Total RNA was prepared from triplicate plates using TRIzol reagent. RNA integrity was determined using an Agilent Bioanalyzer. RNase protection assays were performed using the RPA III kit (Ambion; Austin, TX) and ^32^P-labeled probes generated from the RiboQuant mMyc multiprobe template (BD Biosciences; San Jose, CA) using the T7 MAXIscript kit (Ambion).

### RNA Isolation and Microarray Hybridization for the HOMyc-ER12 Fibroblasts

The experimental conditions for expression profiling of c-Myc target genes induced by 4-hydroxytamoxifen (OHT) in HOMycER12 fibroblasts have been described previously [24]. Genes were clustered according to their expression pattern over time. Unsupervised hierarchical clustering analysis with correlation-based metrics resulted in 9 clusters. Cluster 1 (1,712 probesets) consists of rapidly downregulated c-Myc target genes that remain downregulated for 24 hr. Cluster 2 (1,643 probesets) represents rapidly upregulated genes that remain upregulated. Cluster 3 (616 probesets) contains genes that are quickly downregulated after exposure to OHT then return towards the baseline but remain downregulated at 24 hr. Cluster 4 (178 probesets) represents genes that are quickly upregulated from 1 to 6 hr then fall to baseline. Cluster 5 genes (30 probesets) are rapidly downregulated by OHT from 1 to 6 hr then return towards the baseline by 24 hr. Clusters 6 through 9 had fewer than 5 genes each, so they were not included in the present analyses.

### Identification of c-Myc Targets among Rapamycin Responsive Genes

The dataset of genes that were differentially expressed in response to rapamycin in the WB-F344 and WB311 hepatic cells and the c-Myc target gene dataset in HOMycER12 fibroblasts were compared to search for c-Myc targets in hepatic cells and the fibroblast cell lines. To accomplish this, the ID Converter Database (http://idconverter.bioinfo.cnio.es/) was used to obtain the Ensembl Gene ID and Entrez ID for the list of rapamycin-sensitive genes in the WB-F344 and WB311 hepatic cells and the c-Myc target genes in HOMycER12 fibroblasts. Genes that contained more than one probe ID in the list of differentially expressed genes affected by rapamycin in the WB-F344 and WB311 cells were counted only one time when compared to the existing list of c-Myc targets to avoid overestimating the intersection of the two groups. Therefore, while the total number of Affymetrix probe ID for genes affected by rapamycin in the WB-F344 cells was 2,034, only 1,927 were used for this analysis. While the total number of Affymetrix probe IDs affected by rapamycin in the WB311 cells was 1,236, only 1,130 were used for this analysis.

In addition to the above analysis, the dataset of rapamycin-sensitive genes in the WB-F344 and WB311 cells was aligned with 341 confirmed NFκB target genes previously identified in multiple human cell lines (http://people.bu.edu/gilmore/nf-kb/target/index.html). The purpose of this approach was to perform a second analysis using a gene expression regulating system other than c-Myc to assess the specificity of our findings.

### E-Box Content and Distribution for Rapamycin Responsive Genes in Hepatic Cells

The Affymetrix gene features affected by rapamycin in the WB-F344 and WB311 cells were subjected to further analysis. Promoter regions of annotated genes extending from 3,000 bp upstream to 500 bp downstream of the transcription start sites were extracted from our datasets. The search for E-boxes was restricted to a perfect match with the canonical E-box CACGTG in the promoter sequences [25]. Statistical significance of the results was assessed using a Fisher exact test.

### Profiling of Rapamycin Effect on Gene Expression in Rat Fibroblasts

TGR-1 cells (c-*myc* ^+/+^) and HO15.19 cells (c-*myc* ^−/−^) were plated in 100 mm plates and allowed to attach overnight. Vehicle (DMSO) or 50 nM rapamycin was added for 24 hr. Total RNA was prepared from three independent biological replicates using TRIzol reagent. RNA processing, hybridization and data analysis were carried out at the W. M. KECK Foundation Biotechnology Resources Laboratory at Yale University (New Haven, CT). Gene expression was analyzed using the Illumina RatRef-12 Expression BeadChips and the fluorescence signals were normalized using quantile normalization implemented in BeadStudio (Illumina Inc., San Diego, CA). Illumina data analysis was performed using Partek® software, version 6.3 (Partek Inc., St. Louis, MO). For gene expression comparisons, genes whose change had a level of significance of P>0.01 or fold change less than 1.2-fold were excluded from further analyses. A two-sample t-test using a P-value cutoff of 0.05 with multiple test correction (Benjamini and Hochberg false discovery rate) was applied for each gene to determine if the gene was differentially expressed in the pairwise comparisons of triplicate analyses [26].

### Pathway Analysis

Significantly altered genes from each comparison were mapped to the Kyoto Encyclopedia of Genes and Genomes (KEGG) biopathway database (http://www.genome.ad.jp/kegg/pathway.html). For the Fisher exact test, P-values were employed to determine the enriched gene ontology categories and the overrepresented pathways. The list of differentially expressed genes in each cell line was used for the network reconstruction of pathway analysis, which was performed as described elsewhere [27].

### Data Access and Analysis

The microarray studies described in this paper have been deposited in the NCI's Gene Expression Omnibus (GEO; [28]) and comply with the Minimal Information About a Microarray Experiment (MIAME) standard developed by the MGED Society (http://www.mged.org/Workgroups/MIAME/miame.html). Data derived from the hepatic cell lines may be accessed through the GEO SuperSeries accession number GSE17677 (http://www.ncbi.nlm.nih.gov/geo/query/acc.cgi?acc=GSE17677). Data derived from the rat fibroblast cell lines may be accessed using accession number GSE18845 (http://www.ncbi.nlm.nih.gov/geo/query/acc.cgi?acc=GSE18845).

Chi square analysis was used to determine whether observed frequencies differed from the expected frequencies in the various analyses.

## Results

### Clustering Analysis

**Figure 1:**
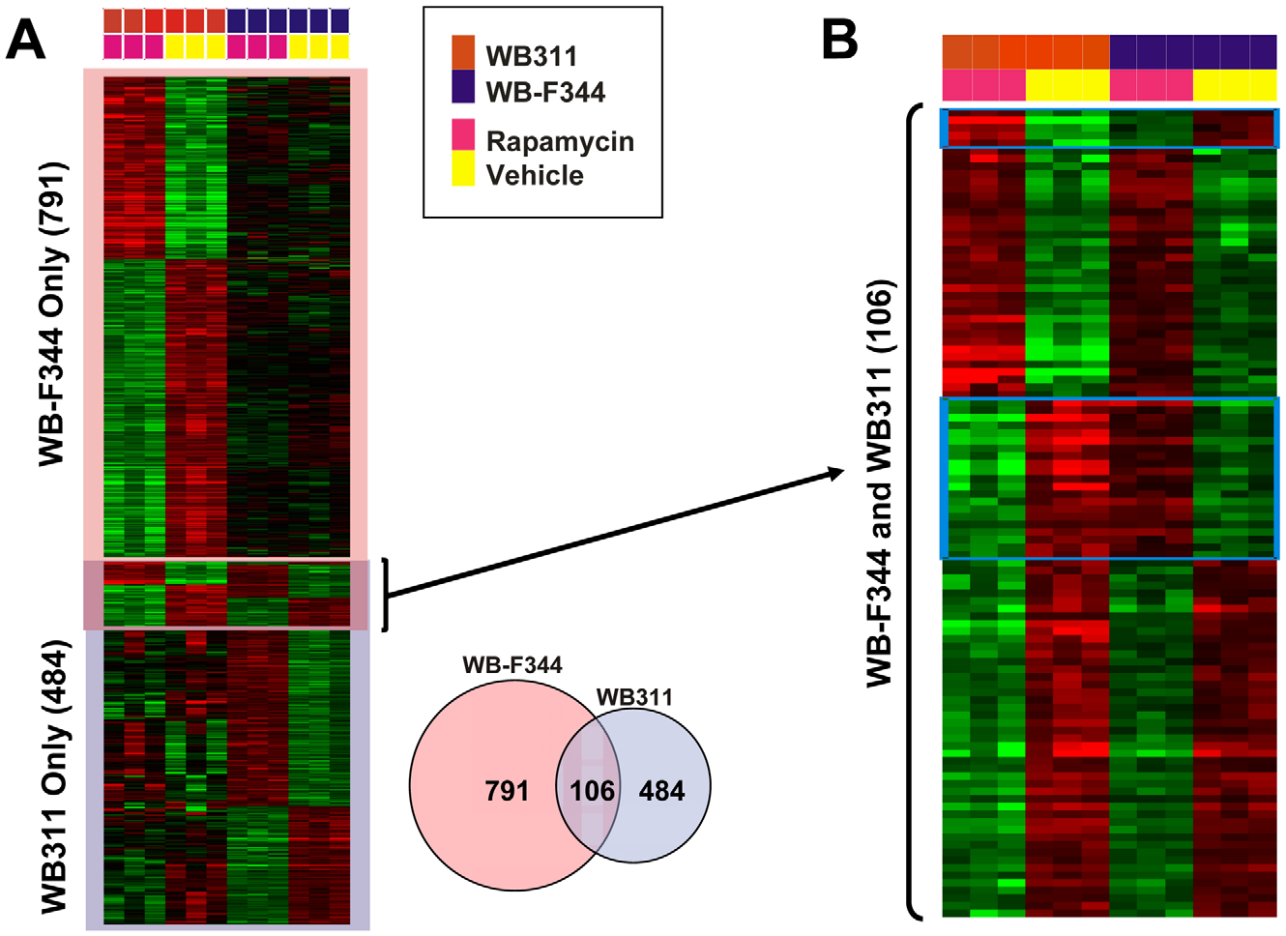
Microarray analysis of the effect of rapamycin on gene expression in hepatic cells. WB-F344 and WB311 cells were exposed to DMSO vehicle or rapamycin (50 nM) for 24 hr. Total RNA was prepared and processed for microarray analysis. *Panel A* shows a clustering analysis and heat map for the expression of all gene features that showed a significant change in response to rapamycin across the four experimental conditions (two cell lines, each plus and minus rapamycin). The accompanying Venn diagram shows the gene features affected in one cell line, the other or in both. *Panel B* shows a magnification of the gene expression pattern of the 106 gene features in the middle box of *Panel A*, representing those that were affected by rapamycin in both cell lines. The blue boxes at the top and in the middle of the heat map represent gene features that were regulated in opposite directions in the WB-F344 and WB311 cells after treatment with rapamycin. The gene features that are not included in the blue boxes represent genes that were co-regulated in the two cell lines.

We previously characterized the gene expression response to rapamycin for the WB-F344 and WB311 hepatic cell lines [18]. Cells were exposed to DMSO vehicle or rapamycin (50 nM) for 24 hr prior to preparation of RNA for microarray analysis. A total of 2,346 gene features showed significant differences across the four experimental groups (2 cell lines, each without or with rapamycin). Hierarchical clustering (Figure 1A) showed that the vehicle control and rapamycin groups segregated for the rapamycin-sensitive WB-F344 cells but not for the rapamycin-resistant WB311 cells. We proceeded to perform a cross-comparison analysis of expression patterns in these two cell lines.

Gene expression ratios were median-centered across the 6 analyses of each cell line. A cross comparison of expression patterns (Figure 1A) showed that 791 gene features were exclusively altered in WB-F344 cells after rapamycin treatment while 484 gene features were exclusively altered in the WB311 cells. Of particular interest were 106 gene features that were affected by rapamycin in both cell lines. Examination of the 106 gene features (Figure 1B) showed that the expression of 26 of the genes was affected in opposite directions in the WB-F344 and WB311 cells. The 80 gene features that were co-regulated in response to rapamycin in the two cell lines (Table S1) were selected for further study. Thirty-three of these genes were upregulated in both cell lines while 47 were downregulated.

### Identification of Overrepresented Transcription Factor Binding Sites in Rapamycin-Regulated Genes in Hepatic Cells

Genes co-regulated by rapamycin in the two cell lines are necessarily regulated independent of growth arrest since the WB311 cells are rapamycin resistant. Our initial hypothesis was that these genes share a common transcriptional control mechanism and, therefore, specific transcription factor binding sites. Of the 80 co-regulated gene features, only 61 had Ensemble IDs and could therefore be recognized by TOUCAN 2. Analysis of the promoter regions of these genes revealed five high-scoring transcription factor matrices that were overrepresented in the designated set of promoters. All five represented binding sites with E-boxes (CACGTG) that corresponded to the consensus sites for the transcription factors Max, MycMax_01, MycMax_02, MycMax_03, and Arnt (Table 1). Nineteen of the 61 genes contained binding sites for these transcription factors (Table 2). Some of these genes contained more than one E-box-containing transcription factor binding in its promoter region. In addition, the binding site that correlated with a specific transcription factor differed in some cases. For example, the transcription factor M00119-V$Max_01 was predicted to bind the motifs TAAT**CACGTG**ATTG and CAAT**CACGTG**ATTA in the promoter of the Eif4b gene while it was predicted to bind the motifs GCAA**CACGTG**ACTC and GAGT**CACGTG**TTGC in the promoter of the Ecm1 gene.

**Table 1:** Overrepresented transcription factor binding sites identified among rapamycin sensitive genes in the WB-F344 and the WB311 hepatic cell lines.

Feature Name	*n* *	*Probability* †	Significance ‡
**M00119-V$MAX_01**	20	1.64E-07	4.363
**M00118-V$MYCMAX_01**	13	2.66E-04	1.154
**M00615-V$MYCMAX_03**	20	4.86E-04	0.892
**M00236-V$ARNT_01**	17	0.001	0.562
**M00123-V$MYCMAX_02**	18	0.003	0.108

* *n* represents the number of times a feature (transcription factor) appeared among the set of gene promoters loaded into the TOUCAN2 program.

† Probability represents the P-value associated with the analysis; p-values less than 0.05 were considered significant and these binding sites were considered to be overrepresented.

‡ Significance is shown here because multiple features (more than one promoter) were interrogated. Positive significance values indicate statistically significant results.

**Table 2:** Genes that are co-regulated in the WB-F344 and WB311 cell lines in response to rapamycin that contain transcription factor binding sites with integral E-boxes.*

Affimetrix		Rapa Effect	Rapa Effect	Transcription Factor	
Identifier	Gene Symbol	WB-F344	WB311	Binding Site Identifier	Transcription Factor Binding Site Sequence
1372124_at	Eif4b_predicted || eukaryotic translation initiation factor 4B	1.42	1.52	M00118-V$MYCMAX_01	TATAATCACGTGATTG
				M00119- V$MAX_01	TAATCACGTGATTG and CAATCACGTGATTA
				M00123-V$MYCMAX_02	ATAATCACGTGATT and AATCACGTGATT and AATCACGTGATT
				M00236-V$ARNT_01	ATAATCACGTGATTGC
				M00615-V$MYCMAX_03	CTATAATCACGTGATTGCCT and AGGCAATCACGTGATTATAG
1385027_at	Transcribed locus	1.60	--	M00118-V$MYCMAX_01	CATCCACGTGCCTG
1379904_at	No Designation	1.39	1.40	M00123-V$MYCMAX_02	CAGCACGCGTGT
				M00236-V$ARNT_01	CGACTCACGTGACGCT
				M00615-V$MYCMAX_03	CTCGACTCACGTGACGCTAC and GTAGCGTCACGTGAGTCGAG
1388698_at	Ecm1 || extracellular matrix protein 1	2.25	1.44	M00119- V$MAX_01	GCAACACGTGACTC and GAGTCACGTGTTGC
				M00123-V$MYCMAX_02	CCGCACGTGTCT and CAACACGTGACT
				M00236-V$ARNT_01	AGAGTCACGTGTTGCC
				M00615-V$MYCMAX_03	CCGGCAACACGTGACTCTAG and CTAGAGTCACGTGTTGCCGG
1387294_at	Sh3bp5 || SH3-domain binding protein 5 (BTK-associated)	1.75	1.28	M00236-V$ARNT_01	GGGACCACGTGAGGTC
1368453_at	Fads2 || fatty acid desaturase 2	-1.64	-1.36	M00123-V$MYCMAX_02	CACCACATGGGA
1385426_at	RGD1305326_predicted || similar to hypothetical protein FLJ20647	-1.45	-1.30	M00118-V$MYCMAX_01	AAACCACGTGGTCA and TGACCACGTGGTTT and CGACCACCTGCAGG
				M00119- V$MAX_01	AAACCACGTGGTCA AND TGACCACGTGGTTT
				M00123-V$MYCMAX_02	GGAAAACCACGTGGTCAATG and CATTGACCACGTGGTTTTCC
				M00615-V$MYCMAX_03	CGAAACCACCGTGGTCAAT and CATTGACCACGTGGTTTCC
1386907_at	Eno3 || enolase 3, beta	-2.54	-1.33	M00118-V$MYCMAX_01	TGTACACGTGCTCG and CGAGCACGTGTACA
				M00119- V$MAX_01	TGTACACGTGCTCG
				M00123-V$MYCMAX_02	GAGCACGTGTAC
				M00236-V$ARNT_01	ATGTACACGTGCTCGA
				M00615-V$MYCMAX_03	ACATGTACACGTGCTCGACT
1389228_at	Similar to RIKEN cDNA 2010309E21 (predicted)	−1.53	−1.26	M00118-V$MYCMAX_01	AACACACGTGGTCG and CGACCACGTGTGTT
				M00119- V$MAX_01	CGACCACGTGTGTT and AACACACGTGGTCG
				M00123-V$MYCMAX_02	GACCACGTGTGT
				M00236-V$ARNT_01	AAACACACGTGGTCGC
				M00615-V$MYCMAX_03	TGAAACACACGTGGTCGCTG
1371332_at	Histone 1, H4a (predicted)	−1.41	−1.29	M00123-V$MYCMAX_02	AACCACATGATA
1367857_at	Fads1 || fatty acid desaturase 1	−1.55	−1.52	M00119- V$MAX_01	TTGTCACGTGTTTC and GAAACACGTGACAA
				M00123-V$MYCMAX_02	AAACACGTGACA
				M00236-V$ARNT_01	TTTGTCACGTGTTTCC
				M00615-V$MYCMAX_03	AAGGAAACACGTGACAAATT and AATTTGTCACGTGTTTCCTT
1383434_at	Transcribed locus	−1.81	−1.38	M00236-V$ARNT_01	TGGATAACGTGTGCGC
1368079_at	Pdk1 || pyruvate dehydrogenase kinase 1	−1.52	−1.41	M00118-V$MYCMAX_01	CTACCACGGTGGTGT and ACACCACGTGGTAG
				M00119- V$MAX_01	CTACCACGTGGTGT and ACACCACGTGGTAG
				M00123-V$MYCMAX_02	CACCACGTGGTA
				MOO615-V$MYCMAX_03	ATCCTACCACGTGGTGTTTC
1372132_at	Cndp2_predicted || CNDP dipeptidase 2 (metallopeptidase M20 family)	−1.90	−1.47	M00119- V$MAX_01	GCACCACGTGTCTC and GAGACACGTGGTGC
				M00123-V$MYCMAX_02	CACCACGTGTCT
				M00236-V$ARNT_01	AGAGACACGTGGTGCG
				M00615-V$MYCMAX_03	TCCGCACCACGTGTCTCTGC and GCAGAGACACGTGGTGCGGA
1375964_at	Psph_predicted || phosphoserine phosphatase (predicted)	−1.89	−1.50	M00123-V$MYCMAX_02	TCGCACGTTGCA
1371445_at	RGD1305092_predicted || similar to ribosome-binding protein p34 - rat	−1.57	−1.29	M00119-V$MAX_01	GTGACACGTGCCTT
				M00236-V$ARNT_01	AGTGACCGTGCCTTC
				M00615-V$MYCMAX_03	CAGAAGGCACGTGTCACTCA and TGAGTGACACGTGCCTTCTG
1377016_at	RGD1310614_predicted || similar to RIKEN cDNA 5730592L21	−1.46	−1.43	M00236-V$ARNT_01	GAGAGCGCGTGACGGC
1375669_at	LOC293702 || similar to binding protein	−1.40	−1.30	M00236-V$ARNT_01	CTAGGCACGTGCACTC and GAGTGCACGTGCCTAG
1367743_at	Pfkl || phosphofructokinase, liver, B-type	−1.39	−1.36	M00119-V$MAX_01	ATGCCACGTGGTTC and GAACCACGTGGCAT
				M00118-V$MYCMAX_01	ATGCCACGTGGTTC and GAACCACGTGGCAT
				M00119-V$MAX_01	ATGCCACGTGGTTC and GAACCACGTGGCAT
				M00123-V$MYCMAX_02	AACCACGTGGCA and AACCACGTGGCA
				M00236-V$ARNT_01	GATGCCACGTGGTTCT and AGAACCACGTGGCATC
				M00236-V$ARNT_01	GATGCCACGTGGTTCT and AGAACCACGTGGCATC
				M00615-V$MYCMAX_03	CCGATGCCACGTGGTTCTCC and GGAGAACCACGTGGCATCGG
				M00615-V$MYCMAX_03	CCGATGCCACGTGGTTCTCC and GGAGAACCACGTGGCATCGG

*Of the 61 co-regulated gene features, 19 were found to have one or more than one E-box-containing transcription factor binding site. For each of these gene features, the fold-change in response to rapamycin, the transcription factor binding site identification and the sequence within the specific gene feature containing that site is shown. “Rapa Effect” denotes the fold-change in response to rapamycin for each individual gene. Note that the fold change in response to rapamycin for 1385027_at could not be determined because two distinct normalization procedures were employed for the clustering versus the differential expression analyses.

### Enrichment of c-Myc Targets in Rapamycin-Modulated Genes in the WB-F344 and WB311 Hepatic Cell Lines

**Figure 2:**
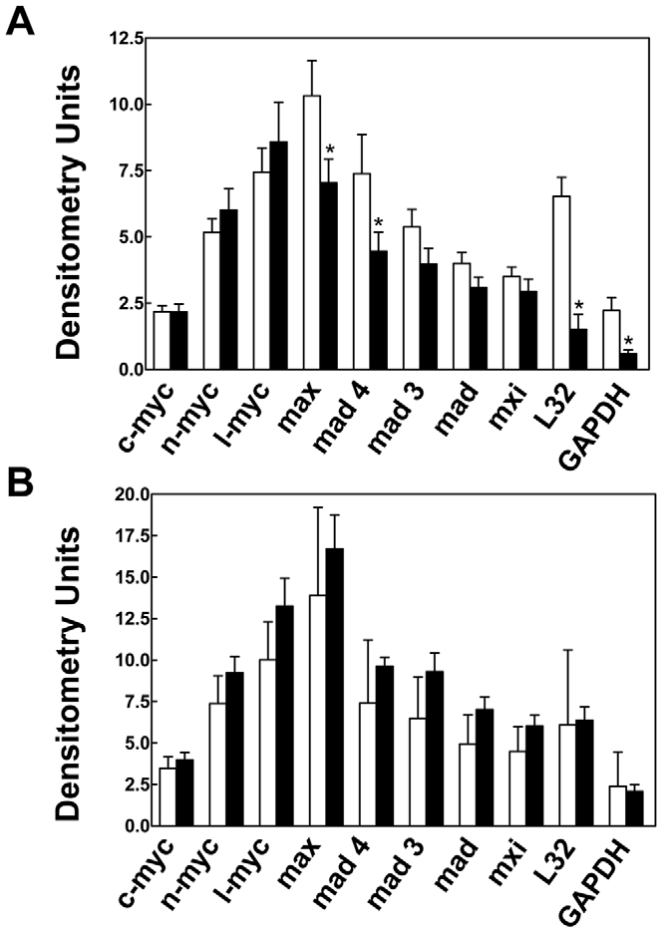
The effect of rapamycin on the expression of components of the Myc signaling network. WB-F344 cells were treated with rapamycin for 6 hr (*Panel A*) or 24 hr (*Panel B*). At the end of the incubation period, total RNA was prepared from triplicate wells for each condition. RNA (21 µg) was analyzed by multiplex RNase protection assay. Results are expressed as the mean + 1SD. The asterisks indicate a significant difference from the control group as determined by unpaired t-test. The densitometry units are arbitrary and should not be used to compare the relative expression of one gene versus another, only the effect of rapamycin. GAPDH, glyceraldehyde 3-phosphate dehydrogenase.

Based on the overrepresentation of E-box-containing transcription factor binding sites among rapamycin-regulated genes, we hypothesized that c-Myc plays a role in the regulation of gene expression by mTOR. Using the WB-F344 cells, we examined the effect of mTOR on members of the Myc signaling network using a multiplex RNase protection assay [29]. Results (Figure 2) showed that rapamycin exposure for 6 hr induced a decrease in the steady state expression of Max and Mad 4. However, the effect was not as marked as the effect on the two genes intended for use as internal standards, ribosomal protein L32 and GAPDH. Of note, members of the Myc family were not affected. At 24 hr, there were no persistent effects on any of the components of the Myc signaling network, L32 or GAPDH. The upregulation of c-*myc* at the longer time point did not reach statistical significance.

**Figure 3:**
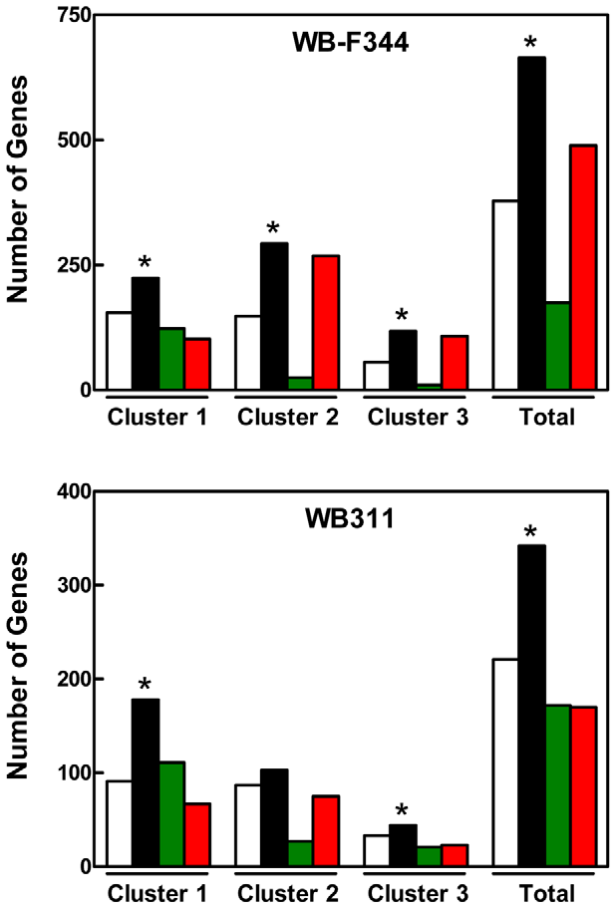
Enrichment of c-Myc targets among rapamycin targets in hepatic cell lines. Microarray results identifying genes affected by rapamycin in WB-F344 cells (*Upper Panel*) and WB311 cells (*Lower Panel*) were compared to c-Myc targets. The latter were identified as genes whose expression was altered in response to c-*myc* induction by OHT in HOMycER12 fibroblasts. The expected (unfilled), observed (black), upregulated (red) and downregulated (green) c-Myc targets regulated in response to rapamycin are shown for the temporal response clusters 1, 2 and 3 in the HOMycER12 fibroblasts and for the total population of genes affected in these cells. *, P<0.05 versus corresponding control were determined by chi square analysis.

We next tested the hypothesis that c-Myc targets would be enriched among the set of mTORC1 targets in both the WB-F344 and WB311 cells. We compared the set of rapamycin-responsive genes in the two hepatic cell lines with a set of c-Myc targets identified in HOMycER12 fibroblasts during c-*myc* induction for up to 24 hr. Results (Figure 3, upper panel) showed that 699 of the 1,927 rapamycin-responsive genes identified in the WB-F344 cells were c-Myc targets. This was significantly greater than the expected number of 377 (P<0.000001). Examination of the distribution of genes among the clusters representing various temporal responses to c-*myc* induction revealed a higher than expected proportion of genes contained in clusters 1 (P<0.000001), 2 (P<0.000001) and 3 (P<0.01).

We performed an analysis to determine if mTORC1 signaling and c-Myc might induce parallel changes in gene expression and that rapamycin effect would reflect this (Figure 3, upper panel). For cluster 1, c-Myc targets that were rapidly and persistently downregulated following c-*myc* induction in the HOMycER12 fibroblasts, there was no difference between the number of c-Myc targets that were upregulated and downregulated in response to rapamycin in the WB-F344 cells. For cluster 2, which represents genes that were upregulated following c-*myc* induction, the majority of c-Myc targets in this cluster (268 of 293 probesets) were downregulated in response to rapamycin. For cluster 3 (genes that were rapidly downregulated then moved towards baseline but remained downregulated at 24 hr following c-*myc* induction), the majority of c-Myc targets (108 of 118 probesets) were downregulated in response to rapamycin. These results indicated limited consistency in the effects of mTORC1 inhibition and c-Myc on the expression of c-Myc target genes.

In the WB311 cell line (Figure 3, lower panel), 342 of 1130 rapamycin-modulated genes were present in the c-Myc target dataset. This number surpassed the expected number of 221, a difference that was significant (P<0.000001). A significant association between rapamycin effect and the effect of c-*myc* induction was confined to clusters 1 and 3. For cluster 1, of the 178 probesets that responded to rapamycin (versus the expected number of 111 probesets), the expression of 111 c-Myc targets was increased while 67 were repressed (P<0.000001). For cluster 3, the observed number of c-Myc targets (44 probesets) exceeded the expected number (37 probesets) (P<0.05). Approximately equal numbers were upregulated and downregulated.

To assess the specificity of the relationship between c-Myc and mTORC1 signaling, we performed a similar analysis using the set of 341 genes that are targets of NFκB. We chose NFκB because, like c-Myc, it is involved in the regulation of a large, diverse set of genes involved in a spectrum of cellular processes [30]. The results of this comparison disclosed that only 27 of the 1,927 rapamycin-responsive genes in the WB-F344 cells were included among the NFκB targets. This number was not significantly greater than the expected number. A similar result was obtained for the WB311 cell line in which only 26 of the 1,130 rapamycin-responsive genes were included in the NFκB dataset.

### E-Box Distribution in the Promoter Regions of Rapamycin-Modulated Genes

**Figure 4:**
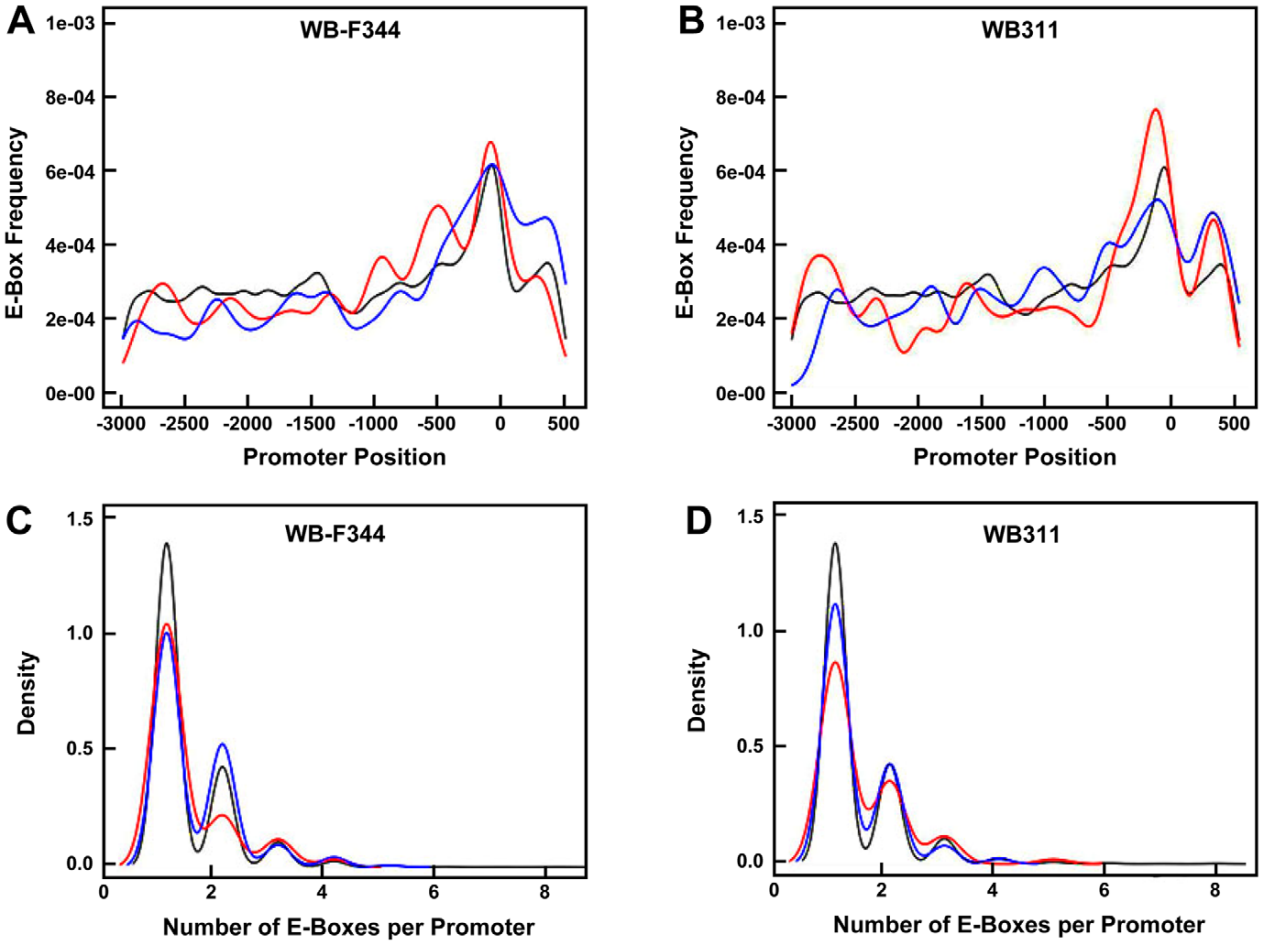
Distribution and number of canonical E-box elements (CACGTG) among rapamycin sensitive genes. Identification of the E-boxes and their distribution was performed as described in the methods section. The promoter sequences spanning −3 kb upstream to +500 bases downstream of the transcriptional start site (TSS) were used in this analysis. Distribution (frequency as a function of position relative to +1, the TSS) of each E-box sequence is shown for WB-F344 cells (*Panel A*) and WB311 cells (*Panel B*). The distribution of the number of E-boxes along the promoters of genes affected by rapamycin is shown for the WB-F344 cells (*Panel C*) and the WB311 cells (*Panel D*). Density (Y-axis) is a probability function representing the normalized frequency of E-boxes. Black lines denote the total number of E-boxes detected in the database, red lines represent E-boxes in the promoters of genes upregulated by rapamycin and blue lines represent the genes downregulated by rapamycin.

We hypothesized that the distribution of E-boxes would differ in the promoter regions of genes that were up-regulated versus down-regulated in response to rapamycin. For the population of rapamycin-responsive genes in the two hepatic cell lines, E-boxes showed a positional bias in that most were located within a region 100 nucleotides upstream of the transcriptional start site (Figures 4A and 4B). There was no apparent difference in the distribution of E-boxes in the two cell lines. In addition, the number of E-box-containing sites among promoters for rapamycin-responsive genes showed similar results for the two cell lines (Figures 4C and 4D). However, the number of promoters with two E-boxes was higher for genes that were downregulated by rapamycin in the WB-F344 cells compared to the genes that were upregulated. This was not the case for the WB311 cells.

### Dependence of Rapamycin Effect on c-Myc in Fibroblasts

To assess the dependence of rapamycin-induced changes in gene expression on c-Myc, we performed studies on two rat fibroblast cell lines, TGR-1 (c-*myc* ^+/+^) and HO15.19 (c-*myc* ^−/−^). Preliminary studies (data not shown) confirmed that the phosphorylation of ribosomal protein S6 (Ser^235/236^) was highly sensitive to inhibition by rapamycin in both cell lines. Studies using ^3^H-thymidine showed that both cell lines were resistant to the growth inhibitory effects of rapamycin (data not shown) based on the criteria that we had established using hepatic cells [18].

We hypothesized that the effect of rapamycin on gene expression would be c-Myc-dependent and that this would be reflected in there being fewer rapamycin-sensitive genes in the HO15.19 fibroblasts (c-*myc* ^−/−^) than in TGR-1 cells (c-*myc* ^+/+^). Both cell lines were exposed to DMSO vehicle or rapamycin (50 nM) for 24 hr. Total RNA was prepared and processed for microarray analysis. Results for the TGR-1 cells showed that 989 gene features (4.5% of the total number on the array) were affected by rapamycin with 521 sequence tags upregulated and 463 downregulated. In the HO15.19 fibroblasts 1,140 probes (5.2% of the total number of gene features on the array) were affected by rapamycin (553 upregulated and 590 downregulated). The number of modulated genes in the HO15.19 fibroblasts was greater than the number in the TGR-1 cells, thus refuting our hypothesis. This conclusion was further supported by the observation that the number of genes showing a 1.5-fold or greater change in expression was higher in the HO15.19 cells than in the TGR-1 cells (data not shown).

**Figure 5:**
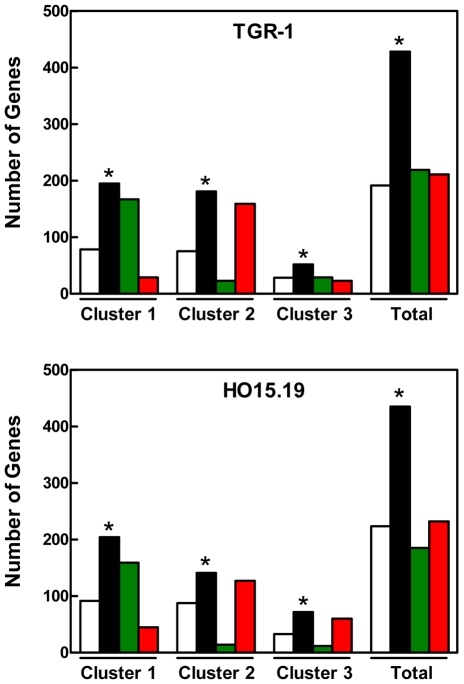
Enrichment of c-Myc targets among rapamycin targets in rat fibroblasts. TGR-1 cells (c-*myc^+/+^*; *Upper Panel*) and c-HO15.19 cells (c-*myc ^−/−^*; *Lower Panel*) were treated with rapamycin (50 nM) for 24 hr. At the end of that time, cells were processed for the preparation of RNA, which was used for microarray analysis. The genes affected by rapamycin were compared to c-Myc targets that were identified as genes whose expression was altered in response to c-Myc induction by OHT in HOMycER12 fibroblasts. The expected (unfilled), observed (black), upregulated (red) and downregulated (green) c-Myc targets regulated in response to rapamycin are shown for the temporal response clusters 1, 2 and 3 in the HOMycER12 fibroblasts and for the total population of genes affected in these cells. *, P<0.05 versus corresponding control were determined by chi square analysis.

Based on the results in hepatic cell lines, we hypothesized that the genes affected by rapamycin in the TGR-1 and HO15.19 cells would show enrichment of c-Myc targets. Results (Figure 5) confirmed this hypothesis in both cells lines. In the TGR-1 cells, 430 of 989 rapamycin modulated genes were in the set of c-Myc targets, far exceeding the expected 192 genes (P<0.000001). As in the hepatic cell lines, the number of rapamycin-sensitive c-Myc targets identified in the two fibroblast lines in clusters 1, 2 and 3 exceeded the number expected.

The direction of regulation in response to rapamycin was examined. For TGR-1 cells (Figure 5, upper panel) cluster 1 genes (downregulated in response to c-*myc* induction), rapamycin induced upregulation of the expression of 167 c-Myc targets while only 29 were downregulated. Among cluster 2 genes (upregulated in response to c-*myc* induction), rapamycin upregulated the expression of 23 c-Myc targets while 159 were downregulated. Similar results were obtained for the HO15.19 cell line (Figure 5, lower panel).

Pathway analysis revealed that rapamycin modulated the expression of genes involved in a number of biochemical and metabolic pathways in both the TGR-1 and HO15.19 fibroblasts (Table 3). These pathways included those involved in steroid, carbohydrate and amino acid metabolism. As was the case for the WB-F344 and WB311 hepatic cells [18], rapamycin affected the glycolysis/gluconeogenesis pathway with phosphoglycerol kinase 1 (Pgk1) being downregulated in both cell lines. This was the only case of a gene being regulated in a manner that determined activity of a pathway in all four cell lines.

**Table 3:** Rapamycin-modulated pathways in TGR-1 and HO15.19 fibroblasts.

TGR-1				
Pathway *	†	P Value ‡	Genes (Total) §	Genes (Significant) ∥
Adipocytokine signaling pathway	▾	0.001	58	9
***Alanine and aspartate metabolism***	▾	0.003	22	5
***Aminoacyl-tRNA biosynthesis***	▾	3.52E-06	28	9
B cell receptor signaling pathway	▾	0.037	52	6
***Bile acid biosynthesis***	▾	0.027	14	3
***Biosynthesis of steroids***	▾	2.27E-07	16	8
Biosynthesis of unsaturated fatty acids	▾	0.039	16	3
***Carbon fixation***	▾	0.006	16	4
Chronic myeloid leukemia	▾	0.004	66	9
Colorectal cancer	▾	0.026	61	7
Complement and coagulation cascades	▾	0.043	54	6
***Fructose and mannose metabolism***	▾	0.0006	23	6
Glioma	▾	0.026	48	6
***Glycine, serine and threonine metabolism***	▾	9.53E-05	32	8
***Glycolysis/Gluconeogenesis***	▾	0.0009	34	7
***Neuroactive ligand-receptor interaction***	▴	0.004	211	2
Non-small cell lung cancer	▾	0.014	42	6
***Olfactory transduction***	▴	2.87E-15	687	1
Pancreatic cancer	▾	0.028	62	7
Pentose phosphate pathway	▾	0.033	15	3
Phenylalanine metabolism	▾	0.022	13	3
Phenylalanine, tyrosine and tryptophan biosynthesis	▾	0.00016	7	4
Prostate cancer	▾	0.019	71	8
Proteasome	▾	0.00025	20	6
Small cell lung cancer	▾	0.012	65	8
Terpenoid biosynthesis	▾	0.013	4	2
TGF-beta signaling pathway	▾	0.04	67	7
Thyroid cancer	▾	0.004	23	5
Urea cycle and metabolism of amino groups	▾	0.0076	17	4
***Valine, leucine and isoleucine degradation***	▾	0.0009	25	6

HO15.19				
Pathway		P Value	Genes (Total)	Genes (Significant)
***Alanine and aspartate metabolism***	▾	0.0044	22	5
Alzheimer's disease	▾	0.037	25	4
***Aminoacyl-tRNA biosynthesis***	▾	4.98E-08	28	11
***Bile acid biosynthesis***	▾	0.032	14	3
***Biosynthesis of steroids***	▾	0.0076	16	4
Bisphenol A degradation	▾	0.015	4	2
***Carbon fixation***	▾	0.046	16	3
Cell cycle	▾	0.0017	89	12
DNA replication	▾	9.31E-05	30	8
ECM-receptor interaction	▾	0.0098	59	8
Focal adhesion	▾	0.018	142	14
***Fructose and mannose metabolism***	▾	0.0054	23	5
***Glycine, serine and threonine metabolism***	▾	0.001	32	7
***Glycolysis/Gluconeogenesis***	▾	0.028	34	5
MAPK signaling pathway	▾	0.0044	201	20
Mismatch repair	▾	0.0095	17	4
***Neuroactive ligand-receptor interaction***	▴	0.0019	211	2
Notch signaling pathway	▾	0.014	39	6
Nucleotide excision repair	▾	0.0091	36	6
***Olfactory transduction***	▴	5.35E-18	687	0
p53 signaling pathway	▾	0.013	50	7
Prion disease	▾	0.012	10	3
Purine metabolism	▾	0.0047	100	12
Pyrimidine metabolism	▾	1.51E-05	55	12
Renal cell carcinoma	▾	0.013	50	7
Ribosome	▾	0.018	53	7
Tyrosine metabolism	▾	0.037	25	4
Valine, leucine and isoleucine biosynthesis	▾	0.004	7	3
***Valine, leucine and isoleucine degradation***	▾	0.008	25	5

* Pathways that were affected by rapamycin in both cell lines are shown in bold italic font.

† The downward and upward pointing arrowheads indicate whether pathways were down- or up-regulated, respectively, in response to rapamycin.

‡ Corrected P-values were calculated using the Fisher exact test.

§ Genes (total), number of genes associated with a given pathway.

∥ Significant genes, number of genes associated with a given pathway that were significantly affected by rapamycin.

**Figure 6:**
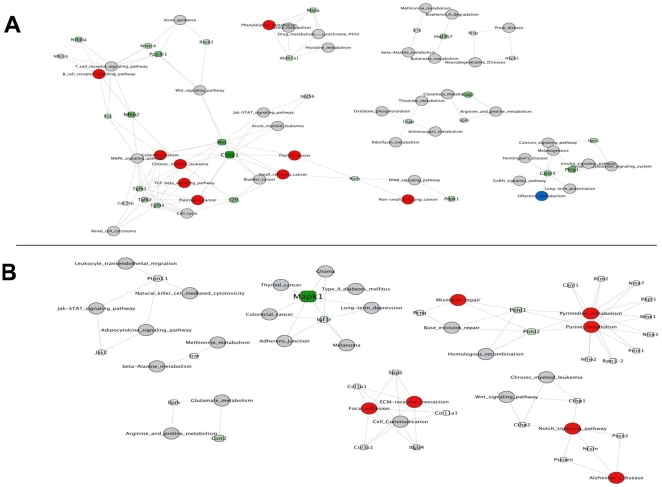
Bipartite network of pathways and genes identified as rapamycin responsive in rat fibroblasts. The microarray analyses described for *Figure 5* were analyzed for networks of pathways that were significantly affected by rapamycin. Pathways are represented by circles and genes by squares. Gray circles indicate non significant pathways. Red and blue circles indicate significant pathways that are overrepresented and underrepresented, respectively. The green tone on the squares indicates the degree of pathways membership (from light green representing genes connected to few pathways to dark green for hubs). *Panel A* shows the pathways and related critical genes affected by rapamycin in TGR-1 cells. *Panel B* shows the pathways affected by rapamycin in HO15.19 cells. The Mapk1 gene appeared as critical but was not connected to any significant pathways. No significant gene was associated with the overrepresented pathways.

Pathway network reconstruction analysis was also performed in order to identify critical genes affected by rapamycin among the interphase of connected pathways. This analysis has already been reported for the WB-F344 and WB311 cells [18]. It showed that two critical genes, c-*myc* and *ccnd1* (cyclin D1) were connected with critical pathways in both cell lines. In the TGR-1 cells treated with rapamycin, a number of overrepresented pathways were similarly interconnected to these two genes (Figure 6A). Both genes were upregulated by rapamycin in the TGR-1 cells. Neither was identified in the pathway network reconstruction analysis for the HO15.19 cell line (Figure 6B), perhaps consistent with the fact that these cells lack c-Myc.

## Discussion

The present studies were undertaken for the purpose of identifying new mechanisms by which signaling via the mTOR pathway regulates gene expression at the transcriptional level. Our starting point was a series of studies on two hepatic cell lines, one that is sensitive to the anti-proliferative effects of rapamycin and one that is not. We reasoned that the set of genes whose expression was modulated in response to rapamycin in both cell lines would represent genes whose control was independent of growth arrest. We further expected that examination of the promoter regions of these genes would allow for the identification of transcription factor binding domains for mTORC1-responsive transcription factors.

The results we obtained were unexpected. The only transcription factor binding sites that were identified contained E-boxes, the consensus sequence of which is CANNTG, with a palindromic canonical sequence of CACGTG [31], [32]. We interpreted this result as indicating that rapamycin-induced inhibition of signaling by mTORC1 might affect transcriptional control by the c-Myc network [25]. Such a conclusion was supported by the commonality in the biological functions of mTOR and c-Myc. Both regulate cell growth, metabolism, gene expression, ribosome biogenesis and protein translation [13], [21], [33], [34].

In order to test the hypothesis that there was a functional intersection between mTORC1 signaling and c-Myc regulation of gene expression, we interrogated the list of rapamycin-sensitive genes for their presence in a population of genes whose expression was regulated in response to the induction of c-*myc* in rat fibroblasts. This approach to identifying c-Myc targets involved not just the detection of changes in expression but also a kinetic expression profiling analysis [24]. By using this approach, we were able to assess not just the intersection between the populations of genes regulated by c-Myc and mTORC1, but also the upregulation versus downregulation of these genes. The results of this analysis demonstrated that c-Myc targets were indeed over-represented among the population of rapamycin-sensitive genes. With regard to direction of regulation, there was a modest concordance that was consistent with co-regulation by c-Myc and mTORC1, two positive regulators of cell growth and proliferation. Furthermore, these observations pertained to both the rapamycin-sensitive and rapamycin-resistant cell lines. The significance of these observations was supported by a similar analysis using a population of genes identified as targets for NFκB. In this case, no relationship with mTORC1 effects was identified.

Based on the intersection between the populations of genes regulated by mTORC1 and c-Myc, we hypothesized that c-Myc might be a direct or indirect participant in the pathway by which mTORC1 regulates gene expression. To test this hypothesis, we characterized rapamycin-induced changes in gene expression in the HO15.19 cell line. These cells were originally derived from TGR-1 cells, which were in turn derived from the Rat-1 fibroblast cell line [35]. HO15.19, made null for c-*myc* by homologous recombination [21], are an appropriate model system for studying the role of c-*myc* in a variety of cell processes. In addition, the TGR-1 and HO15.19 cells represented a second model system in which we were able to confirm the intersection in the regulation of gene expression by mTORC1 and c-Myc.

To further assess the relationship between c-Myc and mTORC1 in the regulation of transcription, we examined the distribution of E-boxes among rapamycin responsive genes in the WB-F344 and WB311 hepatic cells. The significance of E-boxes lies in their role in c-Myc function in that c-Myc regulation of target genes appears to involve direct binding to E-boxes. Mutations in the E-boxes of two c-Myc target genes, nucleolin and BN51, result in the abrogation of transcriptional activation by c-Myc [36]. Mutations in E-boxes are also associated with the removal of the repression of gene activation induced by the c-Myc binding partner Mad [36]. Further, recent studies have shown that upregulated c-Myc targets in the HOMycER12 fibroblasts induced by OHT were enriched for E-boxes (manuscript in preparation, Sedivy et al.). We found that the E-box distribution along the promoter region of rapamycin-regulated genes in the WB-F344 and WB311 cells was similar and that they resembled the distribution observed in drosophila [37] and humans [25]. The E-box distribution in all of these cases tends to show location near the transcription start site. We found that the majority of promoters in rapamycin-regulated genes in the WB-F344 and WB311 cells contained one E-box. We did not find a difference in the number or distribution of E-boxes among genes that were upregulated versus downregulated in response to rapamycin. In summary, our data indicate that c-Myc and mTORC1 may overlap in their regulation of transcription via E-boxes, but that this overlap is independent of c-Myc itself.

Although c-Myc and mTOR are known to be central regulators of comparable biological processes, this is the first study, to our knowledge, that has identified a set of genes that are targets of both c-Myc and mTORC1. mTOR [13], [38] and c-Myc [38] are known to regulate cellular metabolism and, more specifically, glucose metabolism [38], [39]. We found that rapamycin affected various metabolic and biosynthetic pathways in the TGR-1 and HO15.19 cells, similar to our observations in the WB-F344 and WB311 cells. The glycolysis/gluconeogenesis pathway was affected by rapamycin in all four cell lines with the gene for phosphoglycerate kinase-1 (*Pgk1*) being downregulated in every case. Further study would be warranted to determine the significance of this observation.

With regard to the intersection between c-Myc and mTOR signaling, our pathway network reconstruction analyses indicated a role for c*-myc* in mTORC1 action in the WB-F344, WB311 and TGR-1 cell lines. Prior studies have shown that rapamycin can downregulate both the transcription [40] and translation [40]–[42] of c*-myc* in several cell types. It may be that the effect of rapamycin on the expression of *c-myc* and cyclin D is not uniform and is cell type specific. Other studies examining the intersection between c-Myc and mTOR have demonstrated an ability of c-Myc to modulate signaling through the mTOR pathway via transcriptional downregulation of the tuberous sclerosis complex genes [43], [44], which are negative regulators of mTOR-mediated translation activation. Finally, c-Myc has been shown to abrogate the regulation of translation by mTOR by controlling the expression and activity of the key translation regulator, 4EBP-1 [45].

In summary, we have demonstrated an intersection between mTORC1 and c-Myc regulation of gene expression. This was observed in both hepatic cells and fibroblasts. However, control of gene expression by mTORC1 was independent of c-Myc, indicating that the intersection between these two systems is likely a reflection of their biological function, not their mechanism of gene expression control. Furthermore, our inability to detect the involvement of specific transcription factor binding domains in the regulation of gene expression by mTORC1 may indicate the involvement of epigenetic mechanisms, microRNAs or both in mTORC1 signaling.

## Supporting Information

Table S1 Identification of genes that are regulated in both the WB-F344 and WB311 cells in response to rapamycin. The X to the left of a row indicates that the gene is one that was co-regulated in the two cell lines and one for which an Ensembl ID allowed further analysis in Toucan2. Arrowheads indicate the direction of the effect of rapamycin (increase or decrease in expression following exposure to rapamycin). The magnitude of rapamycin effect is given as log-base 2 of the fold-change.
(0.25 MB DOC)
