# Supplementary material for: Regulation of Gene Expression in Hepatic Cells by the Mammalian Target of Rapamycin (mTOR)
Source: PLoS One. 2010 Feb 5;5(2):e9084. doi: 10.1371/journal.pone.0009084 (PMC2816708; doi:10.1371/journal.pone.0009084)
Supplement: Table S1 — Identification of genes that are regulated in both the WB-F344 and WB311 cells in response to rapamycin. The X to the left of a row indicates that the gene is one that was co-regulated in the two cell lines and one for which an Ensembl ID allowed further analysis in Toucan2. Arrowheads indicate the direction of the effect of rapamycin (increase or decrease in expression following exposure to rapamycin). The magnitude of rapamycin effect is given as log-base 2 of the fold-change. (0.25 MB DOC) [file pone.0009084.s001.doc]

|  | **Affymetrix** |  | **WB-F344** |  | **WB311** |  |
| --- | --- | --- | --- | --- | --- | --- |
|  | **Identifier** | **Name** | **Rapa Effect** | **Magnitude** | **Rapa Effect** | **Magnitude** |
|  | 1368342_at | Ampd3 || adenosine monophosphate deaminase 3 | ▲ | 1.018 | ▼ | 0.488 |
|  | 1386994_at | Btg2 || B-cell translocation gene 2, anti-proliferative | ▲ | 0.932 | ▼ | 0.393 |
|  | 1387969_at | Cxcl10 || chemokine (C-X-C motif) ligand 10 | ▲ | 1.007 | ▼ | 0.651 |
|  | 1367581_a_at | Spp1 || secreted phosphoprotein 1 | ▲ | 1.862 | ▼ | 0.513 |
|  | 1368132_at | Tob1 || transducer of ERBB2, 1 | ▲ | 0.688 | ▼ | 0.361 |
|  |  |  |  |  |  |  |
| X | 1382481_a_at | Adam33_predicted || disintegrin and metalloprotease domain 33 | ▲ | 0.604 | ▲ | 0.657 |
| X | 1383075_at | Ccnd1 || cyclin D1 | ▲ | 0.736 | ▲ | 0.556 |
| X | 1371150_at | Ccnd1 || cyclin D1 | ▲ | 0.555 | ▲ | 0.469 |
| X | 1386611_at | Dre1 || Dre1 protein | ▲ | 0.814 | ▲ | 0.852 |
| X | 1383300_at | Dre1 || Dre1 protein | ▲ | 0.718 | ▲ | 0.803 |
| X | 1388698_at | Ecm1 || extracellular matrix protein 1 | ▲ | 1.167 | ▲ | 0.524 |
| X | 1372124_at | Eif4b_predicted || eukaryotic translation initiation factor 4B | ▲ | 0.513 | ▲ | 0.600 |
| X | 1372750_at | Fst || Follistatin | ▲ | 0.758 | ▲ | 0.707 |
|  | 1387788_at | Junb || Jun-B oncogene | ▲ | 0.991 | ▲ | 0.448 |
|  | 1372760_at | LOC499304 | ▲ | 1.004 | ▲ | 0.526 |
| X | 1372585_at | LOC499318 | ▲ | 0.550 | ▲ | 0.309 |
| X | 1375346_at | LOC500993 || similar to hypothetical protein FLJ20010 | ▲ | 0.521 | ▲ | 0.327 |
| X | 1367568_a_at | Mgp || matrix Gla protein | ▲ | 3.779 | ▲ | 0.453 |
| X | 1368308_at | Myc || myelocytomatosis viral oncogene homolog (avian) | ▲ | 1.446 | ▲ | 0.503 |
|  | 1391643_at | No designation | ▲ | 0.445 | ▲ | 0.353 |
|  | 1379904_at | No designation | ▲ | 0.475 | ▲ | 0.491 |
|  | 1376403_at | No designation | ▲ | 1.114 | ▲ | 0.423 |
| X | 1383224_at | Pard6b_predicted || par-6 homolog beta (C. elegans) | ▲ | 0.581 | ▲ | 0.347 |
|  | 1385671_at | PHD finger protein 15 (predicted) | ▲ | 1.003 | ▲ | 0.359 |
| X | 1371974_at | Phyhd1_predicted || phytanoyl-CoA dioxygenase domain containing 1 | ▲ | 0.662 | ▲ | 0.583 |
| X | 1370064_at | Psen2 || presenilin 2 | ▲ | 0.629 | ▲ | 0.729 |
|  | 1388751_at | Rbm24_predicted || RNA binding motif protein 24 (predicted) | ▲ | 0.480 | ▲ | 0.474 |
| X | 1387770_at | RGD:1303168 || putative ISG12(a) protein | ▲ | 0.658 | ▲ | 0.381 |
| X | 1374573_at | RGD1310286_predicted || similar to Dynein 2 light intermediate chain | ▲ | 0.691 | ▲ | 0.371 |
|  | 1381967_at | Rnpc2_predicted || RNA-binding region (RNP1, RRM) containing 2 | ▲ | 0.658 | ▲ | 0.342 |
| X | 1387106_at | Sh3bp4 || SH3-domain binding protein 4 | ▲ | 0.613 | ▲ | 0.364 |
| X | 1387294_at | Sh3bp5 || SH3-domain binding protein 5 (BTK-associated) | ▲ | 0.809 | ▲ | 0.352 |
| X | 1392990_at | Sox17_predicted || SRY-box containing gene 17 | ▲ | 1.503 | ▲ | 0.491 |
| X | 1385027_at | Transcribed locus | ▲ | 0.683 | ▲ | 0.562 |
|  | 1373843_at | Transcribed locus | ▲ | 0.773 | ▲ | 0.888 |
|  | 1374144_at | Transcribed locus | ▲ | 0.749 | ▲ | 0.310 |
|  | 1385641_at | Similar to NP_780521.1 hypothetical protein LOC101148 [Mus musc] | ▲ | 0.704 | ▲ | 0.543 |
|  | 1384380_at | Zinc finger, CCHC domain containing 7 | ▲ | 0.644 | ▲ | 0.352 |
|  |  |  |  |  |  |  |
|  | 1388201_at | Bmp6 || bone morphogenetic protein 6 | ▼ | 0.573 | ▲ | 0.406 |
|  | 1389145_at | Cdc42ep2_predicted || CDC42 effector protein (Rho GTPase binding) 2 | ▼ | 1.144 | ▲ | 0.325 |
|  | 1367940_at | Cmkor1 || chemokine orphan receptor 1 | ▼ | 0.798 | ▲ | 0.535 |
|  | 1370927_at | Col12a1 || procollagen, type XII, alpha 1 | ▼ | 1.633 | ▲ | 0.591 |
|  | 1376105_at | Col14a1_predicted || collagen, type XIV, alpha 1 (undulin) | ▼ | 0.739 | ▲ | 0.755 |
|  | 1368202_a_at | Dab2 || disabled homolog 2 (Drosophila) | ▼ | 0.693 | ▲ | 0.420 |
|  | 1373240_at | Dhrs3_predicted || dehydrogenase/reductase (SDR family) member 3 | ▼ | 0.750 | ▲ | 0.328 |
|  | 1393335_at | Egfl6 || epidermal growth factor-like protein 6 | ▼ | 0.925 | ▲ | 1.100 |
|  | 1369373_at | Fgfr3 || fibroblast growth factor receptor 3 | ▼ | 0.737 | ▲ | 0.418 |
|  | 1391046_at | LOC498295 || similar to SET and MYND domain containing 3 | ▼ | 0.673 | ▲ | 0.556 |
|  | 1370416_at | Mxd3 || Max dimerization protein 3 | ▼ | 0.722 | ▲ | 0.447 |
|  | 1385171_s_at | No designation | ▼ | 1.459 | ▲ | 0.708 |
|  | 1384280_at | Nucleolar and spindle associated protein 1 | ▼ | 0.427 | ▲ | 0.358 |
|  | 1376749_at | Ogn_predicted || osteoglycin (predicted) | ▼ | 1.840 | ▲ | 0.360 |
|  | 1368028_at | Prph1 || peripherin 1 | ▼ | 1.216 | ▲ | 0.368 |
|  | 1389555_at | RGD:1302974 || transcription factor 19 | ▼ | 0.640 | ▲ | 0.356 |
|  | 1367673_at | Selenbp1 || selenium binding protein 2 | ▼ | 0.735 | ▲ | 0.332 |
|  | 1394025_at | Smad6_predicted || MAD homolog 6 (Drosophila) | ▼ | 0.786 | ▲ | 0.368 |
|  | 1378152_at | Transcribed locus | ▼ | 0.834 | ▲ | 0.393 |
|  | 1390536_at | Transcribed locus | ▼ | 1.496 | ▲ | 0.319 |
|  | 1377695_at | Similar to NP_808444.1 hypothetical protein LOC276829 [Mus musc] | ▼ | 0.773 | ▲ | 0.780 |
|  |  |  |  |  |  |  |
| X | 1389349_s_at | 17re || similar to interleukin 17 receptor E isoform 1 | ▼ | 0.658 | ▼ | 0.398 |
| X | 1373928_at | 17re || similar to interleukin 17 receptor E isoform 1 | ▼ | 0.661 | ▼ | 0.365 |
| X | 1370821_at | Cb1-812 | ▼ | 0.720 | ▼ | 0.674 |
| X | 1372132_at | Cndp2_predicted || CNDP dipeptidase 2 (metallopeptidase M20 family) | ▼ | 0.926 | ▼ | 0.556 |
| X | 1369590_a_at | Ddit3 || DNA-damage inducible transcript 3 | ▼ | 1.089 | ▼ | 0.703 |
| X | 1368189_at | Dhcr7 || 7-dehydrocholesterol reductase | ▼ | 0.566 | ▼ | 0.509 |
| X | 1386970_at | Eif2b4 || eukaryotic translation initiation factor 2B, subunit 4 delta | ▼ | 0.483 | ▼ | 0.306 |
| X | 1386907_at | Eno3 || enolase 3, beta | ▼ | 1.349 | ▼ | 0.409 |
|  | 1370281_at | Fabp5 || fatty acid binding protein 5, epidermal | ▼ | 0.678 | ▼ | 0.586 |
| X | 1367857_at | Fads1 || fatty acid desaturase 1 | ▼ | 0.624 | ▼ | 0.601 |
| X | 1368453_at | Fads2 || fatty acid desaturase 2 | ▼ | 0.721 | ▼ | 0.441 |
| X | 1371332_at | Histone 1, H4a (predicted) | ▼ | 0.497 | ▼ | 0.363 |
| X | 1375669_at | LOC293702 || similar to binding protein (LOC293702) | ▼ | 0.488 | ▼ | 0.378 |
| X | 1381474_at | LOC302492 || similar to mCHCR | ▼ | 0.935 | ▼ | 0.640 |
| X | 1367612_at | Mgst1 || microsomal glutathione S-transferase 1 | ▼ | 0.498 | ▼ | 0.427 |
|  | 1368020_at | Mvd || mevalonate (diphospho) decarboxylase | ▼ | 0.754 | ▼ | 0.391 |
|  | 1398618_s_at | No designation | ▼ | 0.943 | ▼ | 0.732 |
|  | 1388647_at | No designation | ▼ | 0.505 | ▼ | 0.466 |
|  | 1389973_a_at | No designation | ▼ | 0.418 | ▼ | 0.424 |
| X | 1367847_at | Nupr1 || nuclear protein 1 | ▼ | 1.119 | ▼ | 0.793 |
| X | 1368079_at | Pdk1 || pyruvate dehydrogenase kinase 1 | ▼ | 0.604 | ▼ | 0.494 |
| X | 1367743_at | Pfkl || phosphofructokinase, liver, B-type | ▼ | 0.484 | ▼ | 0.444 |
| X | 1375964_at | Psph_predicted || phosphoserine phosphatase (predicted) | ▼ | 0.922 | ▼ | 0.587 |
| X | 1372462_at | RGD:1359366 || similar to acetyl CoA transferase | ▼ | 0.744 | ▼ | 0.606 |
| X | 1374524_at | RGD:1359514 || selenocysteine lyase | ▼ | 0.638 | ▼ | 0.475 |
| X | 1374575_at | RGD:1359613 || cAMP responsive element binding protein 3-like 1 | ▼ | 1.263 | ▼ | 0.429 |
| X | 1371542_at | RGD:1359623 || similar to Tubulin alpha-4 chain (Alpha-tubulin 4) | ▼ | 0.523 | ▼ | 0.370 |
| X | 1374906_at | RGD:1359693 || similar to RIKEN cDNA 2810428C21 | ▼ | 0.668 | ▼ | 0.454 |
| X | 1371445_at | RGD1305092_predicted || similar to ribosome-binding protein p34 - rat | ▼ | 0.646 | ▼ | 0.372 |
| X | 1385426_at | RGD1305326_predicted || similar to hypothetical protein FLJ20647 | ▼ | 0.537 | ▼ | 0.381 |
| X | 1374333_at | RGD1306058_predicted || similar to RIKEN cDNA 1110007C09 | ▼ | 0.851 | ▼ | 0.330 |
| X | 1372156_at | RGD1307423_predicted || similar to RIKEN cDNA 1810014L12 | ▼ | 0.758 | ▼ | 0.405 |
| X | 1372966_at | RGD1310174_predicted || hypothetical LOC298504 (predicted) | ▼ | 0.643 | ▼ | 0.336 |
| X | 1377016_at | RGD1310614_predicted || similar to RIKEN cDNA 5730592L21 | ▼ | 0.550 | ▼ | 0.513 |
| X | 1367668_a_at | Scd2 || stearoyl-Coenzyme A desaturase 2 | ▼ | 1.572 | ▼ | 1.134 |
| X | 1388695_at | Shmt2_predicted || serine hydroxymethyl transferase 2 (mitochondrial) | ▼ | 0.505 | ▼ | 0.382 |
| X | 1389228_at | Similar to RIKEN cDNA 2010309E21 (predicted) | ▼ | 0.616 | ▼ | 0.332 |
| X | 1388996_at | Similar to RIKEN cDNA 3110052N05 (predicted) | ▼ | 0.635 | ▼ | 0.438 |
| X | 1372326_at | Slc2a3 || facilitated glucose transporter) member 3 | ▼ | 0.712 | ▼ | 0.396 |
| X | 1393706_at | Steap_predicted || six transmembrane epithelial antigen of the prostate | ▼ | 0.594 | ▼ | 0.480 |
| X | 1367603_at | Tpi1 || Similar to Tpi1 protein | ▼ | 0.465 | ▼ | 0.472 |
| X | 1380410_at | Transcribed locus | ▼ | 0.735 | ▼ | 0.635 |
| X | 1383434_at | Transcribed locus | ▼ | 0.852 | ▼ | 0.470 |
| X | 1397248_at | Transcribed locus | ▼ | 0.898 | ▼ | 0.540 |
| X | 1385926_at | Transcribed locus | ▼ | 0.752 | ▼ | 0.604 |
| X | 1386321_s_at | Trib3 || tribbles homolog 3 (Drosophila) | ▼ | 0.922 | ▼ | 0.984 |
| X | 1385428_at | Xpo4_predicted || exportin 4 (predicted) | ▼ | 0.615 | ▼ | 0.397 |
